# Evaluation of “international transfer-out” among foreign-born pulmonary tuberculosis patients in Japan – what are the implications for a cross-border patient referral system?

**DOI:** 10.1186/s12889-018-6273-0

**Published:** 2018-12-07

**Authors:** Lisa Kawatsu, Akihiro Ohkado, Kazuhiro Uchimura, Kiyohiko Izumi

**Affiliations:** Department of Epidemiology and Clinical Research, the Research Institute of Tuberculosis, 3-1-24, Matsuyama Kiyose, Tokyo, Japan

**Keywords:** Tuberculosis, Migration, Treatment outcome, Patient care, Surveillance

## Abstract

**Background:**

Tuberculosis (TB) patients crossing borders pose a serious challenge to global TB control efforts. The objectives of our study were firstly, to evaluate the trend and size of foreign-born pulmonary TB patients, who had been notified and initiated treatment in Japan but have transferred out of the country while still on treatment; and secondly, to conduct a detailed analysis of these patients and identify possible risk factors for international transfer-out, and discuss policy implications for a cross-border patient referral system for foreign-born TB patients in Japan.

**Methods:**

We conducted a cross-sectional study whereby aggregated cohort data of pulmonary TB cases newly notified to the Japan TB Surveillance system between 1 January 2011 and 31 December 2015 were analyzed. Multinomial logistic regression analysis was conducted to identify and compare the risk factors for international transfer-out.

**Results:**

Among the 668 foreign-born patients whose treatment outcome had been evaluated as “transferred- out”, 51.3% has in fact moved to outside Japan between 2011 and 2015. The proportion of such international transfer-out of total foreign-born patients who had transferred out has more than doubled during the study period, from 23.3% in 2011 to 57.7% in 2015. Some of the risk factors for international transfer-out were being a full-time worker (Relative risk [RR] 2.86, 95% confidence interval [CI] 2.04, 3.99), being diagnosed within 0 to 2 years of arriving to Japan (RR 8.78, 95% CI 4.30,17.90) and within 3 to 5 years (RR 7.53, 95% CI 3.61, 15.68), sputum smear positive (RR 1.95, 95% CI 1.53, 2.48), and coming from Indonesia (RR 1.86, 95% CI 1.13, 3.03).

**Conclusions:**

Providing continuity of care for mobile population is one of the keys to achieving the WHO’s End TB Strategy targets for 2030, and results of our study indicate that a cross-border referral system should be an integral part of TB control among foreign-born persons in Japan.

**Electronic supplementary material:**

The online version of this article (10.1186/s12889-018-6273-0) contains supplementary material, which is available to authorized users.

## Background

The ever increasing movement of people across national borders has given rise to two particular issues in tuberculosis (TB) control; the first being the need for early case detection and diagnosis of TB being brought in from abroad, and the second, the need to ensure continuity of care for patients who have decided to move to another country while still on treatment. In response to the first, several countries have adopted screening programmes specifically targeting immigrants by means of chest-X ray, sputum examinations, tuberculin skin test or interferon-gamma assay [1, 2]. On the other hand, establishing a system of cross-national patient referral, one of the possible solutions to addressing the second issues, has proved much more challenging [3].

Japan is a TB middle-burden country with 17,625 newly notified patients in 2016, giving a notification rate of 13.9 per 100,000. Although the proportion of foreign-born persons among the total cases is relatively small compared to similarly industrialized countries, at 7.9% (*n* = 1338) in 2016, between 2007 and 2016, both the number and the proportion have steadily increased approximately by 2.3 times, from 3.5% (*n* = 842) to 7.9% (*n* = 1338), and that among those aged 15 to 24 by 2.5 times, from 23.8% (*n* = 231) to 58.6% (*n* = 471) [4].

In principle, 95% of the cost of TB treatment is covered by public subsidy for outpatients, and 100% for patients needing hospitalization, regardless of nationality. Directly Observed Treatment and patient education are provided in hospital settings for hospitalized patients, and in the community, generally by public health center (PHC) nurses, for outpatients. PHCs are local government bodies which are responsible for receiving notifications from physicians who have diagnosed TB, registering them onto the national TB surveillance system, the Japan Tuberculosis Surveillance (JTBS), and managing treatment support for the patients (but not providing treatment). Yet several studies on treatment outcome and adherence behavior of foreign-born TB patients at national and sub-national level have indicated poorer performance as compared with Japan-born patients, with high proportions of those who have been “lost to follow-up” and have “transferred-out” [5, 6].

The current Japan Tuberculosis Surveillance (JTBS) system evaluates the treatment outcome as recorded by the PHC which initially registered the patient. Thus, when the patient moves out of its local jurisdiction, his/her treatment outcome is recorded as “transferred-out”. For domestic transfer-out, whereby patients move within Japan, most patients are usually transferred-in and continue and complete their treatment [7]. However, as for international transfer-out, whereby patients move out of Japan, there is currently no standardized system or procedures for referring foreign-born patients to medical institutions in the country of their origin. Cross-national patient referral has been organized on several occasions but on an ad-hoc basis, and treatment outcome is usually not followed-up [8].

Considering that foreign-born persons moving both in and out of Japan are likely to continue to increase, and also the growing threat of drug-resistant TB due in sufficient patient care, the potential impact of international transfer-out cannot be underestimated. Yet to date, no study has attempted to evaluate the size of foreign-born patients transferring out of Japan while still on TB treatment. In this study, we thus firstly sought to evaluate the trend and size of international transfer-out among the foreign-born pulmonary TB (pTB) patients, who had been notified to the JTBS and who had initiated treatment in Japan. Secondly, we attempted a detailed analysis of those who have transferred out of Japan, and identify possible risk factors for international transfer-out, and discuss whether there are any policy implications for a cross-border patient referral system for foreign-born TB patients in Japan.

## Method

We conducted a cross-sectional study whereby aggregated cohort data of pTB cases newly notified to the JTBS between 1 January 2011 and 31 December 2015 were analyzed. Those whose country of birth was recorded as “unknown” were excluded from the analysis.

Under the JTBS, treatment outcome of drug-susceptible pTB patients is evaluated at the end of 12 months. The outcome categories include “cured”, “treatment completed”, “died”, “treatment failure”, “lost to follow-up”, “transferred out”, “still on treatment”, and “unevaluated” [4]. “Transferred out” includes both transfer within and to outside Japan, and are not disaggregated in the analysis of treatment outcome. A more detailed information regarding the system, and various definitions can be found elsewhere [4]. In evaluating the size of “international transfer-out”, we thus turned to another variable of the JTBS, namely, the “reason for de-registering” from the TB registry. PHC which initially received the notification de-registers the patient from its TB registry when he or she has been transferred and in doing so, is asked to enter the reason for de-registering the patient from the following options; “follow-up no longer required”, “died due to TB”, “died due to non-TB cause”, “not diagnosed as TB”, “domestic transfer-out”, “international transfer-out”, “re-registered as either new active TB case or LTBI during follow-up” and “others”. We thus firstly disaggregated those who have been evaluated as “transferred out” under treatment outcome category by “reason for de-registering”, to evaluate the actual and proportional size of “international transfer-out”, and its trend during the study period.

Secondly, we attempted a detailed analysis of those foreign-born patients who have transferred outside Japan, by examining and comparing their socio-demographic characteristics with foreign-born patients who have successfully completed treatment, and those who have been lost to follow-up, and those who have transferred outside of Japan. For the purpose of our analysis, those whose treatment outcome was recorded as “cured”, “treatment completed”, and “transferred out” but whose reason for being de-registered was domestic transfer-out, were combined as “treatment success”. We then conducted a multinomial logistic regression analysis, taking “treatment success”, “international transfer-out” and “lost to follow-up” as outcomes, to identify possible risk factors for international transfer-out.

Based on previous studies, and the practical availability from the JTBS dataset, the following were chosen as independent variables; sex, age groups, occupational category (regrouped into “high school and university students”, “full-time employed workers”, “temporary and day workers”, “unemployed” and “others”), number of years between entry to Japan and being diagnosed as TB in Japan, sputum smear examination result, and countries of birth. We also examined the timing at which patients had moved out of Japan by counting the day from when treatment was initiated to when the patient was de-registered. The R version 3.1.3 (R Development Core Team, Vienna, Austria) was used for all statistical analyses.

## Results

Size and trend of “international transfer-out” among foreign-born pTB patients.

**Fig. 1:**
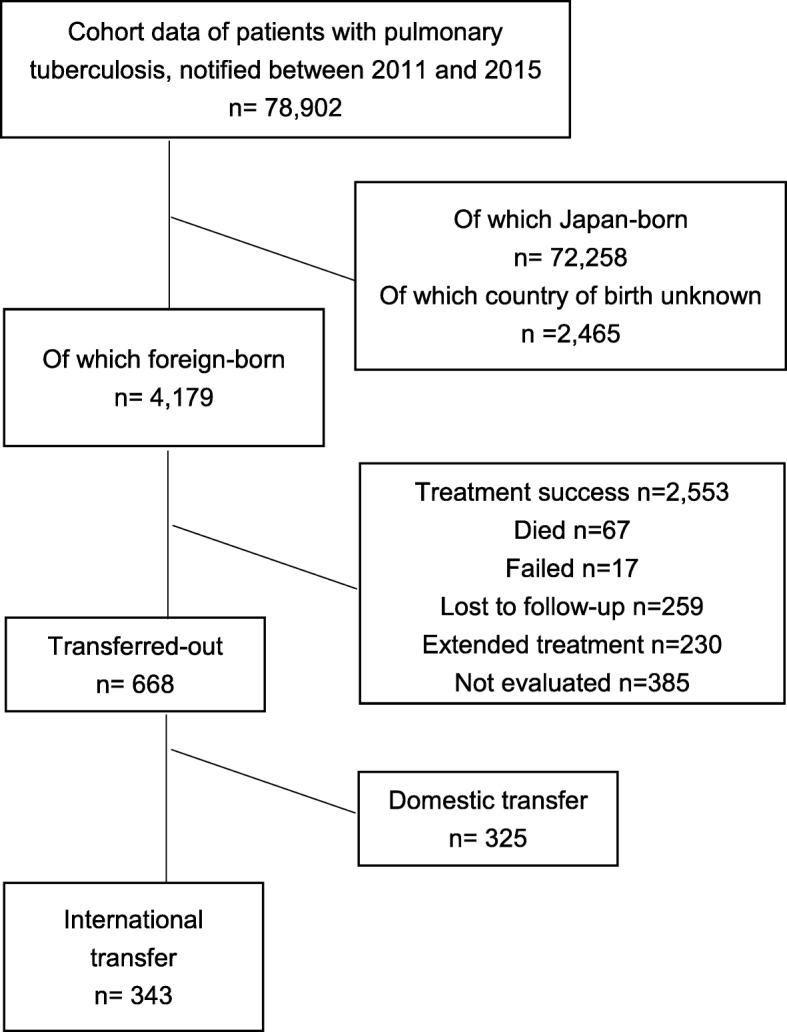
Flow-chart of the study population

Flow-chart of the study population is shown in Fig. 1. The treatment outcome was known for 4179 foreign-born patients who had been notified to the JTBS between 2011 and 2015, and is summarized in Table 1, by age groups. Of the total 4179 foreign-born patients, 668 had been evaluated as “transferred out”. Analysis of the reason for deregistering from the TB registry among the 668 foreign-born patients who had been evaluated as “transferred out” revealed that 51.3% (*n* = 343) has in fact moved to outside Japan, with the highest proportion of international transfer-out among those between aged 15 and 24 years old (10.9%, 145/1330).

**Table 1:** Treatment outcomes of foreign-born pTB patients by age groups, 2011–2015

	0–14		15–24		25–54		55+		Total	
	n	%	n	%	n	%	n	%	n	%
Completed	16	76.2	589	44.3	1217	49.7	115	30.3	1937	46.4
Cured	0	0.0	217	16.3	345	14.1	54	14.2	616	14.7
Died	0	0.0	0	0.0	13	0.5	54	14.2	67	1.6
Failed	0	0.0	3	0.2	9	0.4	5	1.3	17	0.4
Lost to follow-up	0	0.0	67	5.0	169	6.9	23	6.1	259	6.2
Not evaluated	3	14.3	110	8.3	233	9.5	39	10.3	385	9.2
Still on treatment	0	0.0	51	3.8	140	5.7	39	10.3	230	5.5
International transfer-out	1	4.8	145	10.9	165	6.7	32	8.4	343	8.2
Domestic transfer-out	1	4.8	148	11.1	158	6.5	18	4.7	325	7.8
Total	21	100.0	1330	100.0	2449	100.0	379	100.0	4179	100.0

*pTB* pulmonary tuberculosis

**Fig. 2:**
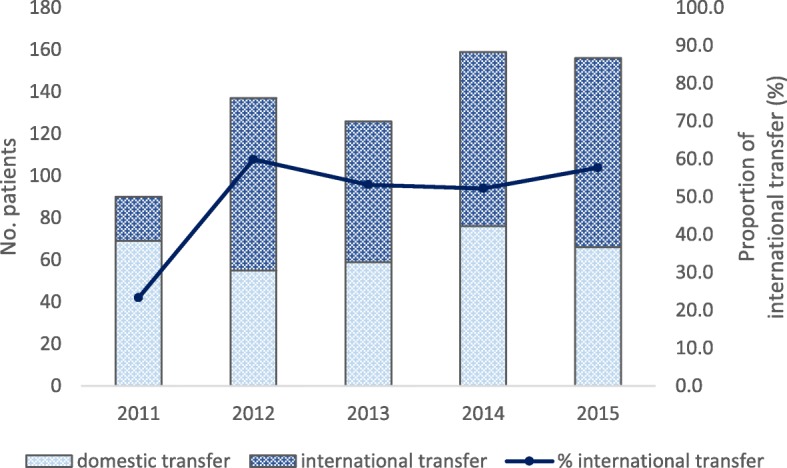
Foreign-born patients who have been evaluated as “transfer-out”, by type of transfer, 2011–2015 *n* = 668)

The proportion of international transfer out of total foreign-born patients who had been evaluated as “transferred out” has more than doubled during the study period, from 23.3% in 2011 to 57.7% in 2015, as shown in Fig. 2 (see also Additional file 1).

Characteristics of foreign-born pTB patients who have transferred-out.

The socio-demographic characteristics of foreign-born patients who have transferred outside of Japan (*n* = 343) are summarized and compared with those lost to follow-up (*n* = 259), and those who have successfully completed treatment in Japan (i.e. “completed”, “cured”, and “domestic transfer-out”, *n* = 2878), in Table 2. Among those who have transferred out, the proportion of males and of those aged between 0 and 24 years old were higher as compared to those who were lost to follow-up and those who have successfully completed treatment (59.25 vs 42.1 and 47.8%, 42.6% vs 25.9,33.7%). Among those who have transferred out, the largest occupational category was “full-time workers” (42.6%), followed by “unemployed” (21.3%). However, among those lost to follow-up and those who have successfully completed treatment, the largest group was similarly “full-time workers” (36.7 and 33.9%), but followed by “high school and university students” (22.8 and 30.0%). Among those who have transferred out, 40.8% had arrived in Japan within 2 years of being diagnosed, while the proportion was much smaller among those lost to follow-up and those who have successfully completed treatment (22.4 and 24.1%). The proportion of smear positive upon diagnosis was also higher among those who have transferred out, in contrast to those lost to follow-up and those who have successfully completed treatment (52.2% vs 22.0 and 38.1%). Finally, while China and the Philippines together contributed to approximately half of the country of birth for all treatment outcome groups, the proportion of those from Indonesia was notably higher among those who have transferred out, than those lost to follow-up and those who have successfully completed treatment (11.1% vs 3.1 and 4.9%).

**Table 2:** Characteristics of foreign-born patients who have transferred outside of Japan, who have been lost to follow-up, and who have successfully completed treatment, 2011–2015

	International transfer-out		Lost to follow-up		Treatment success	
	n	%	n	%	n	%
Total	343	100	259	100	2878	100
Sex						
Male	203	59.2	109	42.1	1376	47.8
Female	140	40.8	150	57.9	1502	52.2
Age groups (years)						
0–24	146	42.6	67	25.9	971	33.7
25–54	165	48.1	169	65.3	1720	59.8
54-plus	32	9.3	23	8.9	187	6.5
Occupational status						
High school and university students	65	19.0	59	22.8	864	30.0
Full-time workers	146	42.6	95	36.7	977	33.9
Temporary employed/day workers	37	10.8	28	10.8	302	10.5
Unemployed	73	21.3	54	20.8	467	16.2
Others, including unknown	22	6.4	23	8.9	268	9.3
Time between entry to Japan and diagnosis (years)						
0–2	140	40.8	58	22.4	694	24.1
3–5	67	19.5	35	13.5	364	12.6
5–10	5	1.5	19	7.3	213	7.4
10+	9	2.6	36	13.9	301	10.5
Unknown	122	35.6	111	42.9	1306	45.4
Sputum smear						
Negative	158	46.1	194	74.9	1742	60.5
Positive	179	52.2	57	22.0	1096	38.1
Not done/Unknown	6	1.7	8	3.1	40	1.4
Country of birth						
Others	53	15.5	46	17.8	533	18.5
China	115	33.5	72	27.8	810	28.1
Indonesia	38	11.1	8	3.1	142	4.9
Korea	15	4.4	15	5.8	172	6.0
Nepal	7	2.0	15	5.8	218	7.6
Philippines	65	19.0	70	27.0	695	24.1
Thailand	8	2.3	12	4.6	63	2.2
Vietnam	42	12.2	21	8.1	245	8.5

### Risk factors for “international transfer-out”

Table 3 summarizes the results of the multinomial logistic regression analysis, taking “treatment success” as reference. Among those who have transferred out, males were at a higher risk of transferring out compared with females (relative risk [RR] 1.54, 95% confidence interval [CI]1.21,1.97). Similarly, taking students as reference, being a full-time worker (RR 3.01, 95% confidence interval [CI] 2.14, 4.23), temporary worker (RR 2.84, 95% CI 1.78, 4.54), unemployed (RR 3.40, 95% CI 2.21, 5.22) and others (RR 2.34, 95% CI 1.34, 4.10) were identified as risk factors for international transfer-out. As for time between entry to Japan and diagnosis, taking “more than 10 years” as reference, those patients who had been diagnosed within 0 to 2 years (RR 8.78, 95% CI 4.30, 17.90), within 3 to 5 years (RR 7.53, 95% CI 3.61, 15.68), and unknown (RR 3.70, 95% CI 1.84, 7.43) were at a significant risk of transferring out of Japan. Taking negative result for sputum smear examination, a positive result (RR 1.95, 95% CI 1.53, 2.48), and coming from Indonesia (RR 1.86, 95% CI 1.13, 3.03) were also identified as risk factors. On the other hand, being aged between 25 and 54 years old (RR 0.71, 95% CI 0.54–0.93) and coming from Nepal (RR 0.28 95% CI 0.12, 0.63) were identified as protective factors for international transfer-out.

**Table 3:** Relative risks for international transfer-out and lost to follow-up

	International transfer-out			Lost to follow-up		
	Relative Risk	95% CI	*P* value	Relative Risk	95% CI	*P* value
Sex						
Female	Reference					
Male	1.54	1.21–1.97	< 0.01	0.83	0.63–1.09	0.18
Age groups (years)						
0–24	Reference					
25–54	0.71	0.54–0.93	0.01	1.32	0.94–1.86	0.11
54+	1.10	0.65–1.85	0.72	1.74	0.95–3.18	0.07
Occupational status						
High school and university students	Reference					
Full-time workers	3.01	2.14–4.23	< 0.01	1.33	0.90–1.95	0.15
Temporary employed/day workers	2.84	1.78–4.54	< 0.01	1.29	0.77–2.16	0.33
Unemployed	3.40	2.21–5.22	< 0.01	1.58	0.99–2.52	0.06
Others, including unknown	2.34	1.34–4.10	< 0.01	1.19	0.68–2.08	0.54
Time between entry to Japan and diagnosis (years)						
10+	Reference					
0–2	8.78	4.30–17.90	< 0.01	0.93	0.57–1.51	0.76
3–5	7.53	3.61–15.68	< 0.01	1.00	0.59–1.67	0.90
5–10	0.91	0.30–2.79	0.87	0.79	0.44–1.44	0.44
Unknown	3.70	1.84–7.43	< 0.01	0.79	0.52–1.19	0.26
Sputum smear						
Negative	Reference					
Positive	1.95	1.53–2.48	< 0.01	0.42	0.31–0.57	< 0.01
Not done/Unknown	1.55	0.62–3.85	0.35	1.83	0.84–3.99	0.13
Country of birth						
Others	Reference					
China	1.32	0.91–1.89	0.14	1.11	0.75–1.66	0.60
Indonesia	1.86	1.13–3.03	0.01	0.66	0.30–1.46	0.31
Korea	0.78	0.42–1.46	0.44	0.94	0.50–1.75	0.84
Nepal	0.28	0.12–0.63	< 0.01	1.02	0.54–1.90	0.96
Philippines	0.90	0.60–1.34	0.60	1.07	0.71–1.61	0.74
Thailand	1.40	0.62–3.12	0.42	2.19	1.08–4.41	0.03
Vietnam	1.53	0.96–2.43	0.07	1.11	0.63–1.94	0.72

*CI* confidence interval

None of the variables which had been identified as risk factors for international transfer-out were significant factors for lost to follow-up. In fact, the only statistically significant variable which was identified for lost to follow-up was a sputum smear examination result, but with relative risk pointing towards the opposite direction – in other words, being smear positive was identified as a protective factor (RR 0.42, 95% CI 0.31, 0.57).

### Timing of transfer-out

**Fig. 3:**
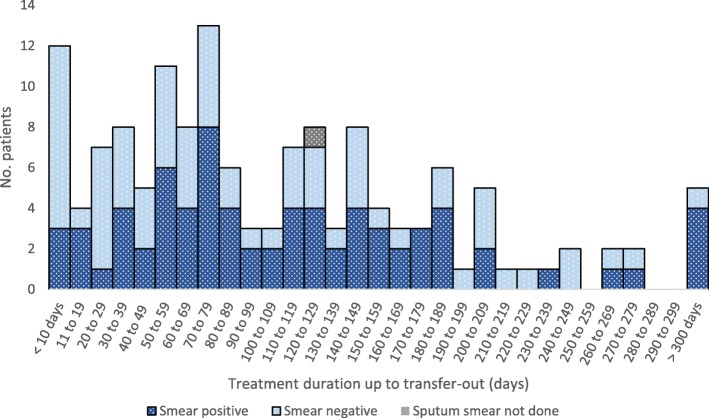
Treatment duration up to transfer-out, in days

Out of 343 foreign-born patients who had transferred out of Japan, the treatment duration was known for 142. Figure 3 shows that while 66.9% 95/142) had transferred out after 60 days, 33.1% 47/142) did so in their intensive phase. Furthermore, 8.5% *n* = 12) had transferred out within 10 days of being notified see also Additional file 2). Of the 47 who transferred out in their intensive phase, 40.4% *n* = 19) were sputum smear positive, indicating that they may have moved out while they were still being hospitalized i.e. before confirmation of negative conversion.

## Discussion

This study is the first ever attempt to have focused specifically on foreign-born TB patients transferring out of Japan while still on treatment for TB at a national level. Our results have shown that over the study period, 8.2% 343/4179) of the total foreign-born pTB patients had moved out of Japan during their treatment, and their final treatment outcomes were unknown. Those with some sort of work, those unemployed, those who have recently arrived in Japan, those smear positive and those coming from Indonesia were at a higher risk of transferring out of Japan.

The results are reasonable in that compared with students, employed persons are more likely to have the financial capacity to pay for their travel back to their home country. A recent study on the economic situation of foreign-born students in Japan has indicated that of the total respondents, as much as 74.8% had to work outside the school hours to maintain their living, and that 33.5% were working more than 20 h a week [9]. Thus, even if they had wanted to, it would probably be difficult for many to travel back to their countries of origin. Employed persons, on the other hand, would be in a position to choose from a wider selection of options, including completing their treatment in their home country. This also explains the differences by countries of origin – for example, the proportion of those from Indonesia entering with student visa is relatively small compared with other nationalities, at approximately 15% [10]. The majority arrive to Japan with some sort of employment or internship visa, and are therefore more likely to be able to return to their country for completing TB treatment, should they wish to do so. On the other hand, approximately 60% of those from Nepal enter Japan as students, many of whom are students of private Japanese language schools and training colleges, and are thus self-funded [10, 11].

Those who have recently arrived in Japan are more likely to face linguistic, cultural and social difficulties in continuing the treatment in Japan. Inadequate medical and welfare support for foreign-born patients is increasingly raised as a serious issue and has been pointed out in numerous studies [12–14]. In a similar study on treatment outcome of foreign-born TB patients in Osaka, time of entry to Japan within 5 years of being diagnosed was also identified as a risk factor for international transfer-out [6]. Furthermore, in Japan, patients who have tested positive for sputum smear examination are required to be hospitalized until they are proved no longer infectious. It is thus quite understandable that sputum-smear positive foreign-born patients who are told that they need to be hospitalized face even greater anxiety, and feel less confident about continuing their treatment in Japan.

Interestingly, sputum smear positive result was identified as a protective factor for lost to follow-up – however, this is consistent with a study on the risk factors for lost to follow-up in Japan. The authors have argued that the perceived “seriousness” of the disease on part of the patients, together with intensive patient education they receive during hospitalization, may make these patients less prone to treatment interruption [15]. Perhaps a detailed qualitative analysis should follow to explore why some smear positive foreign-born patients decide to return to their home country, while others decide to stay in Japan to complete their treatment. What our results do indicate is that a different approach is needed to address international transfer-out, to that required for foreign-born patients becoming lost to follow-up in Japan.

It is certainly not the intention of the authors to argue that these patients should complete their treatment in Japan, or that the option of completing the treatment in Japan is better than that of returning to their home country. What is an issue is that when foreign-born patients decide to move out of Japan, such cross-border referral is not conducted systematically and their ultimate treatment outcomes remain unknown. Several studies have shown that mobility is a significant risk factor for treatment interruption [16], and consequently for development of drug resistance [17, 18].

Though still limited, several countries have begun to address the issue of continuation of care for patients leaving their country. In the Netherlands, its Tuberculosis Control Plan 2016–2020 clearly states that concerted effort to ensure that TB patients who leave the Netherlands during their treatment are referred to treatment providers in the destination countries, and KNCV Tuberculosis Foundation began monitoring and documenting the outcomes of this process [3]. Within Europe, the European Respiratory Society and the WHO Regional Office for Europe have also jointly developed a web-based, open-access multi-lingual system, TB Consilium, with a function to introduce a patient to a treatment provider in another country [19]. The United States Cure TB Program, which is a federally funded program by San Diego TB Control Program and now an official partner with the Center for Disease Prevention and Control, has been systematically providing binational United States and Mexico) referral services since 1997, and has recently expanded their program to cover the rest of the world [20, 21]. Another non-profit organization, Migrant Clinicians Network, has also been serving mobile patients with health needs since 1996. Their services include transfer of medical records, follow-up and other patient support, and as for TB, has consistently reported treatment completion rate of more than 84% [22]. Furthermore, a recent modelling study has indicated that a scale-up of these cross-border referral services would not only benefit the patients themselves, but also the Unites States directly, as well as the international community at large [20]. Results of our study indicate that such a cross-border referral system is necessary in Japan too–a small pilot project with countries with relatively large number of returning patients, such as China, the Philippines and Vietnam, may be considered.

This study is not without limitations. Firstly, the analysis of timing of transfer-out was only possible for slightly less than a half of those patients who had been recorded as “international transfer-out”. Furthermore, the claim that patients have moved out of Japan is not in any way confirmed by, for example, airplane tickets or official documents, and is based on self-report. It is thus quite possible that a patient, having informed the public health center of his or her intention of going back to country of origin, in fact continues to stay in Japan. Secondly, the data analyzed only covered drug-susceptible pTB patients – a detailed analysis of patients with multi-drug resistance, as well as extrapulmonary TB, ought to follow. The strength of this study is that it uses the data from a national surveillance system, with relatively high data entry rate [4]. Furthermore, data quality is assured via a variety of mechanisms including the system’s internal verification program [4] – it is thus reasonable to claim credibility of the data and generalizability of our results.

## Conclusions

Unigonorable number of foreign-born TB patients are moving out of Japan while still on treatment, with their final treatment outcome unknown. Some of them were sputum smear positive, and had moved shortly after being diagnosed. These patients present immediate concern of the risk of causing secondary infection, and of themselves interrupting treatment, as well as mid-to long-term concern of exacerbating anti-microbial resistance in the world. It is without doubt that providing continuity of care for mobile population is one of the keys to achieving the WHO’s End TB Strategy targets for 2030, and it is about time Japan gets its act together in building a cross-border referral system for foreign-born patients who are diagnosed in Japan. Such a system can be started out as a small pilot project with countries to which relatively large number of patients are returning, such as China, the Philippines and Vietnam, and be expanded gradually.

## Additional files

Additional file 1: Trend in the number and proportion of international transfer-out, 2011–2015. A table showing the number of domestic and international transfer out. (XLSX 11 kb)

Additional file 2: Timing of international transfer-out. A table showing the number of days between treatment initiation and de-registration from TB registry, of foreign-born patients who have transferred outside Japan, by sputum smear examination result. (XLSX 14 kb)
